# Supplementary material for: Centrifugation-Assisted Solid-Phase Extraction Coupled with UPLC-MS/MS for the Determination of Mycotoxins in ARECAE Semen and Its Processed Products
Source: Toxins (Basel). 2022 Oct 29;14(11):742. doi: 10.3390/toxins14110742 (PMC9697234; doi:10.3390/toxins14110742)
Supplement: Supplementary file 1 [file toxins-14-00742-s001.zip › toxins-1973186-supplementary.pdf]

Supplementary Materials

Centrifugation-Assisted Solid-Phase Extraction Coupled with UPLC-MS/MS for the Determination of Mycotoxins in ARECAE Semen and Its Processed Products

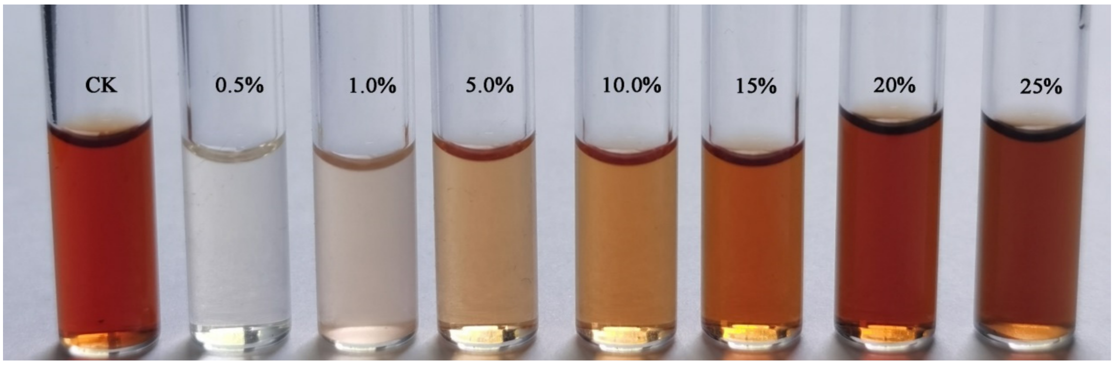

Figure S1. Effect of different concentrations of acetic acid on the color of SPE purified solution.

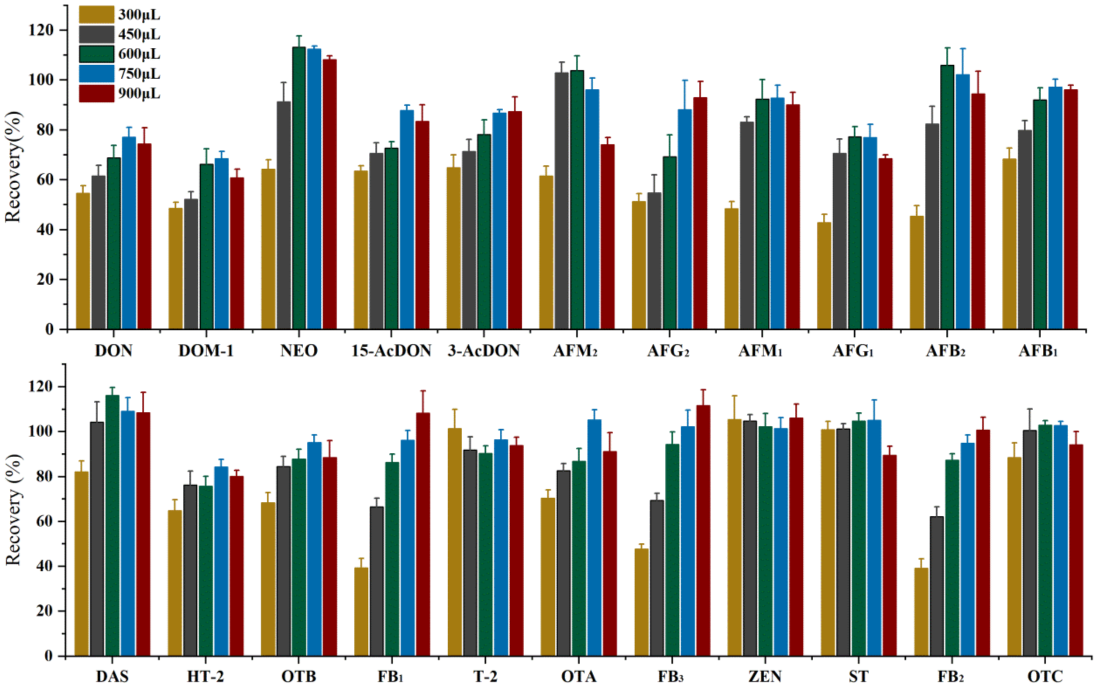

Figure S2. Effect of SPE loading volumes on the recoveries of selected mycotoxins.

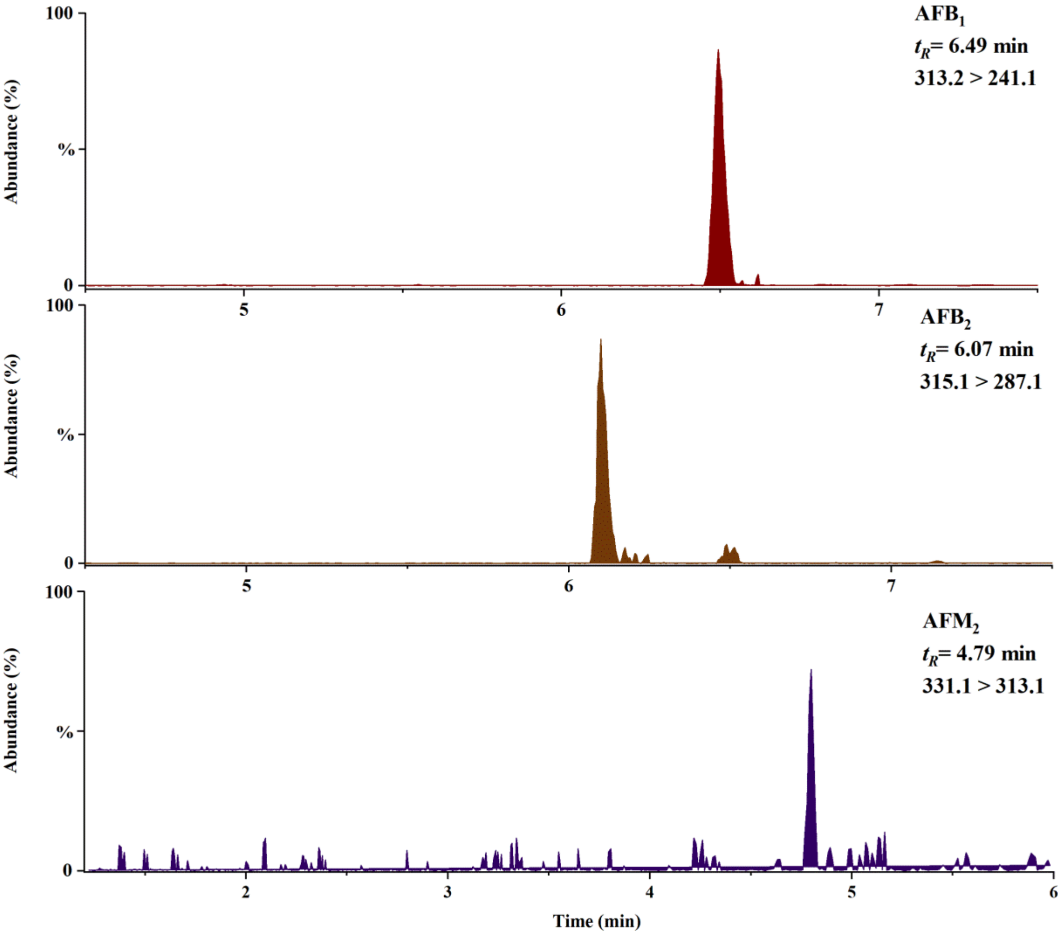

Figure S3. MRM chromatograms of positive WBL-4 sample

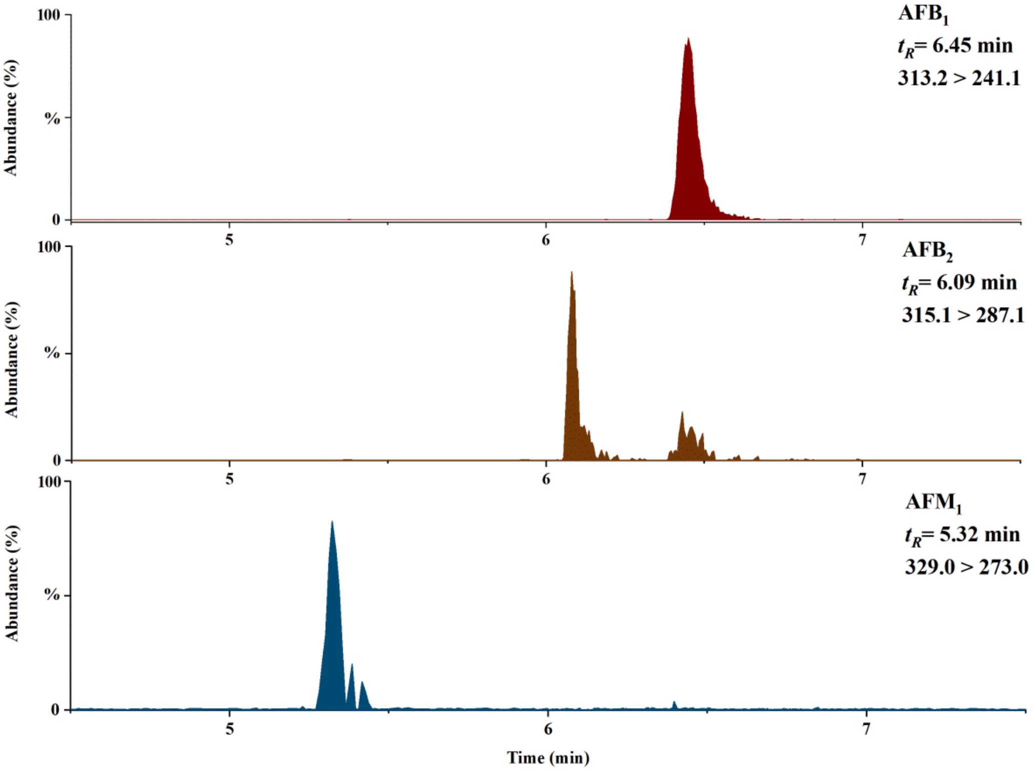

Figure S4. MRM chromatograms of positive BLS-2 sample

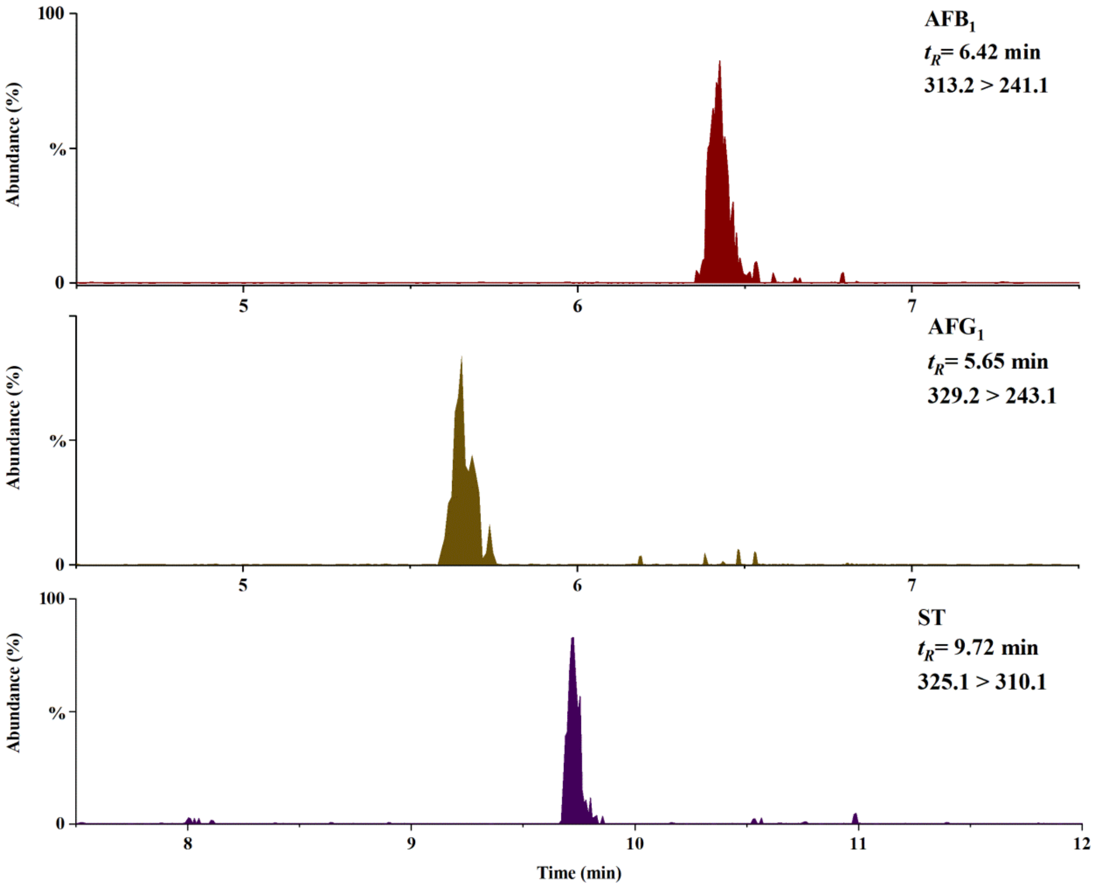

Figure S5. MRM chromatograms of positive BLS-15 sample

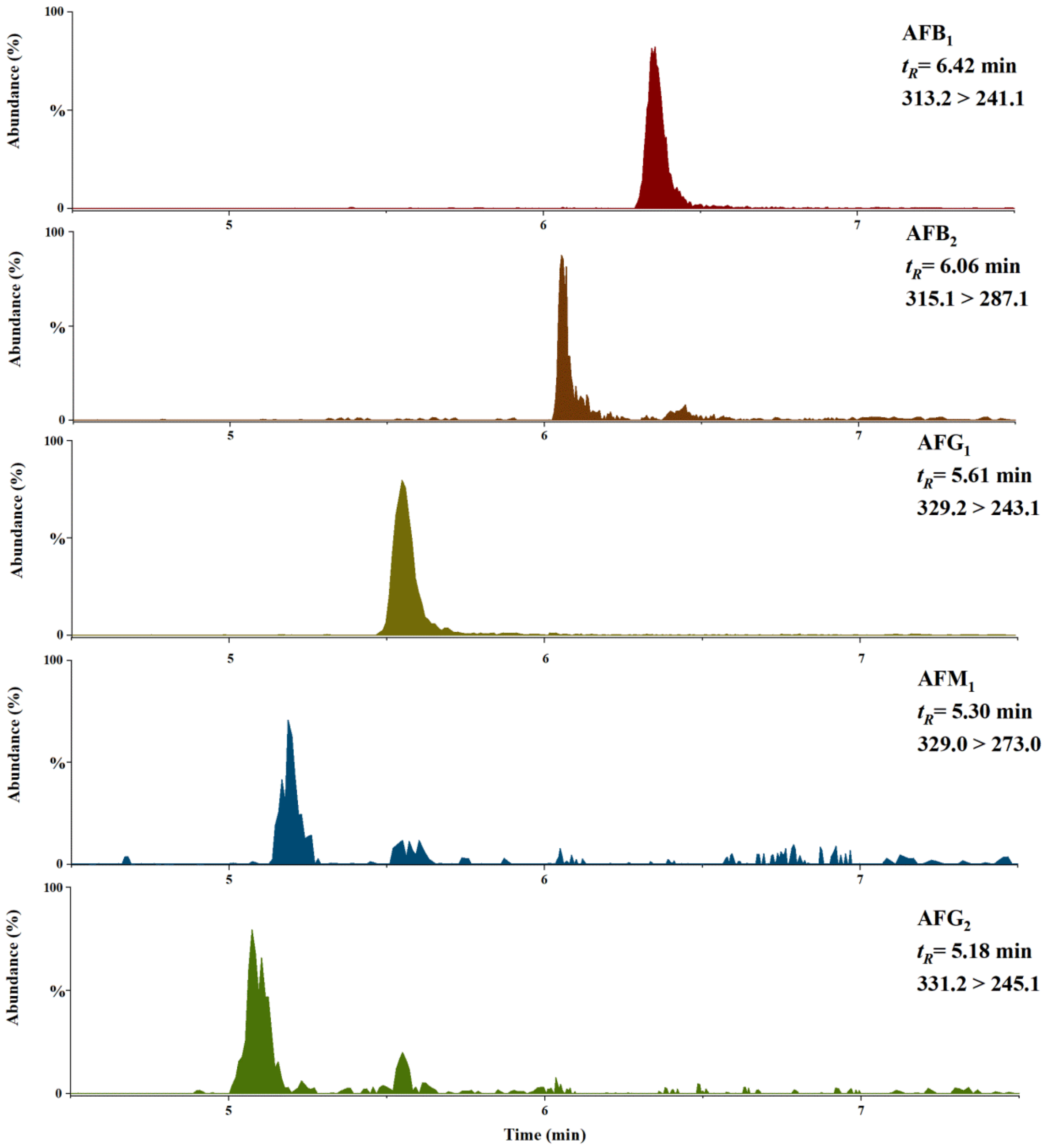

Figure S6. MRM chromatograms of positive JBL-15 sample

Table S1. Recoveries of selected mycotoxins in WBL, JBL and DFP

Mycotoxins	WBL		JBL		DFP	
	Recovery (%)	RSD (%)	Recovery (%)	RSD (%)	Recovery (%)	RSD (%)
DON	72.87	8.09	67.45	7.41	70.88	8.74
DOM-1	73.46	8.43	70.04	5.76	65.33	9.42
NEO	103.42	9.78	108.44	10.73	112.87	7.76
3-AcDON	89.36	5.77	92.14	7.56	93.87	3.93
15-AcDON	84.05	5.85	94.33	4.68	103.49	7.16
AFM_2	91.06	5.47	88.67	3.84	90.67	7.28
AFG_2	90.81	6.59	82.45	7.15	79.58	3.09
AFM_1	86.04	3.52	88.39	5.78	84.67	5.47
AFG_1	74.16	6.44	72.61	8.42	75.38	12.37
AFB_2	96.14	3.76	95.08	3.86	92.76	8.94
AFB_1	103.60	4.28	107.21	4.36	111.87	7.44
DAS	107.99	13.87	102.59	9.82	109.43	10.84
HT-2	77.93	6.08	84.42	6.53	92.69	8.67
OTB	96.49	4.38	88.86	6.25	94.51	3.64
FB_1	88.54	11.73	90.08	5.29	81.35	6.85
T-2	95.56	3.79	89.74	6.58	93.27	4.09
OTA	95.79	8.72	99.07	9.03	94.23	5.13
FB_3	84.79	12.19	87.46	10.54	79.59	9.45
ZEN	90.77	5.47	105.74	8.53	103.43	3.38
ST	99.53	4.39	103.46	5.57	98.82	4.19
FB_2	96.37	6.24	98.89	8.11	77.58	8.49
OTC	106.24	7.68	103.87	11.67	96.54	7.23

Spike level: 5.0 μg/kg for AFB_1, AFB_2, AFG_1, AFG_2, AFM_1, and AFM_2; 25.0 μg/kg for OTA,OTB, and OTC; 50.0 μg/kg for DON, DOM-1, HT-2, T-2, ST, ZEN, 15-Ac DON, 3-Ac DON NEO, and DAS; and 125.0 μg/kg for FB_1, FB_2, and FB_3
